# Supplementary material for: Dose-dependent and strain-dependent anti-obesity effects of Lactobacillus sakei in a diet induced obese murine model
Source: PeerJ. 2019 Mar 21;7:e6651. doi: 10.7717/peerj.6651 (PMC6431538; doi:10.7717/peerj.6651)
Supplement: Supplemental Information 2 — Detailed analytical composition of the low-fat (LFD) and high-fat (HFD) diets used in this study. (A) Low fat diet (38057; Purina Laboratory Rodent Diet); (B) High fat diet (D12492; Research Diets Inc., New Brunswick, NU, USA). [file peerj-07-6651-s002.docx]

**Supplementary information**

**Table S1**

**Dose dependent and strain-dependent anti-obesity effects of *Lactobacillus sakei* in a diet induced obese murine model**

Yosep Ji^1*^, Young Mee Chung^2*^, Soyoung Park^1*^, Dahye Jeong^2^, Bongjoon Kim^2^, Wilhelm H. Holzapfel^1^

^1^Department of Advanced Green Energy and Environment, Handong Global University, Pohang, Gyungbuk 37554, South Korea

^2^Beneficial microbes center, CJ Foods R&D, CJ CheilJedang Corporation, Suwon-si, South Korea

**Supplementary Table S1.** Detailed analytical composition of the low-fat (LFD) and high-fat (HFD) diets used in this study. (A) Low fat diet (38057; Purina Laboratory Rodent Diet); (B) High fat diet (D12492; Research Diets Inc., New Brunswick, NU, USA).

| **A. Low fat diet (LFD; Purina Laboratory Rodent Diet 38057)** | | | | | | |  |
| --- | --- | --- | --- | --- | --- | --- | --- |
| Nutrients | | Calories (%) | | | | |  |
|  |  |  | Fat (ether extract) 12.41%, Protein 24.52%, Carbohydrate 63.07%  Total 3,940 Kcal/Kg | | | | |
| Protein g (%) | 20 g% | Arginine 1.26 g%, Cystine 0.37 g%, Glycine 0.87 g%,  Histidine 0.5 g%, Isoleucine 0.82 g%, Leucine 1.47 g%,  Lysine 1.01 g%, Methionine 0.33 g%, Phenylalanine 0.98 g%,  Tyrosine 0.63 g%, Threonine 0.72 g%,  Tryptophan 0.25 g%, Valine 0.91 g% | | | | |  |
| Fat g (%) | 4.5 g% | Linoleic Acid 1.10 g%, Linolenic Acid 0.12 g%,  Arachidonic Acid 0.02 g%, Omega-3 Fatty Acids 1.11 g% | | | | |  |
| Fiber g (%) | 3.7 g% | Fiber (Crude) 3.7 g% | | | | |  |
| Minerals | - | Ash 7.25 g%, Calcium 1.2 g%, Phosphorus 0.62 g%,  Phosphorus (non-phytate) 0.4 g%, Potassium 0.82 g%,  Magnesium 0.16 g%, Sulfur 0.22 g%, Sodium 0.34 g%,  Chlorine 0.47 g%, Fluorine 21.38 ppm, Iron 112.93 ppm,  Zinc 128.85 ppm, Manganese 95.49 ppm,  Copper 22.74 ppm, Cobalt 0.76 ppm, Iodine 1.42 ppm,  Chromium 0 ppm, Selenium 0.32 ppm | | | | |  |
| Vitamins | - | Vitamin K 6.69 ppm, Thiamin Hydrochloride 11.02 ppm,  Riboflavin 11.57 ppm, Niacin 217.7 ppm,  Pantothenic Acid 88.72 ppm, Choline Chloride 3447.96 ppm,  Folic Acid 13.6 ppm, Pyridoxine 11ppm,  Biotin 0.15 ppm, B12 41 ppm, Vitamin A 28.03 IU/kg,  Vitamin D3 (added) 4 IU/kg, Vitamin E 100 IU/kg | | | | |  |
|  |  |  | | | | |  |
|  |  |  |  |  |  |  | |

**B. High fat diet (HFD; Research Diets** **D12492) (Research Diets Inc., New Brunswick, NU, USA)**

|  | | | | | | |
| --- | --- | --- | --- | --- | --- | --- |
| Calories (%) | | Ingredients (g) | | | | |
|  |  | Protein | Fat | Carbohydrate | Fiber | Minerals/  Vitamins/  Dye |
| Fat | 60.00% | Casein, Lactic, 30 Mesh 200g  Cystine, L 3g | Lard 245g  Soybean Oil, USP 25g | Lodex 10 3g  Sucrose, Fine Granulated 72.8g | Solka Floc, FCC200 50g | S10026B 50g  Choline Bitartrate 2g  V10001C 1g  Dye, Blue FD&C #1, Alum. Lake 35-42% 0.05g |
| Carbohydrate | 20.00% |  |  |  |  |  |
| Protein | 20.00% |  |  |  |  |  |
| Total | 100% | 203 | 270 | 197.8 | 50 | 53.05 |
|  |  | 773.85 (g) | | | | |
