# Supplementary material for: Dose-dependent and strain-dependent anti-obesity effects of Lactobacillus sakei in a diet induced obese murine model
Source: PeerJ. 2019 Mar 21;7:e6651. doi: 10.7717/peerj.6651 (PMC6431538; doi:10.7717/peerj.6651)
Supplement: Supplemental Information 3 — Effect of the test materials/strains on body weight in HFD-induced obese mice. LFD, low-fat diet; HFD, high-fat diet; CJB38, CJB46 and CJLS03 denote the three Lactobacillus sakei strains; the three dose levels of each strain administered together with the HFD were 1 X 1010 CFU/mL (high-dose, H), 1 X 109 (medium-dose, M) and 1 X 108 CFU/mL (low-dose, L). Data expressed as mean ± SD (n=10). *, **: significantly different from ND group at p<0.05, p<0.01; #, ##:: significantly different from HFD group at p<0.05, p<0.01. [file peerj-07-6651-s003.docx]

**Supplementary information**

**Table S2**

**Dose dependent and strain-dependent anti-obesity effects of *Lactobacillus sakei* in a diet induced obese murine model**

Yosep Ji^1*^, Young Mee Chung^2*^, Soyoung Park^1*^, Dahye Jeong^2^, Bongjoon Kim^2^, Wilhelm H. Holzapfel^1^

^1^Department of Advanced Green Energy and Environment, Handong Global University, Pohang, Gyungbuk 37554, South Korea

^2^Beneficial microbes center, CJ Foods R&D, CJ CheilJedang Corporation, Suwon-si, South Korea

**Supplementary Table S2.** Effect of the test materials/strains on body weight in HFD-induced obese mice. LFD, low-fat diet; HFD, high-fat diet; CJB38, CJB46 and CJLS03 denote the three *Lactobacillus* *sakei* strains; the three dose levels of each strain administered together with the HFD were 1 X 10^10^ CFU/mL (high-dose, H), 1 X 10^9^ (medium-dose, M) and 1 X 10^8^ CFU/mL (low-dose, L). Data expressed as mean ± SD (n=10).

^*, **^: significantly different from ND group at *p*<0.05, *p*<0.01; ^#, ##:^: significantly different from HFD group at *p*<0.05, *p*<0.01.

| Group | | Day 0 | Day 4 | Day 7 | Day 10 | Day 14 | Day 17 | Day 21 | Day 24 | Day 28 | Day 31 | Day 35 | Day 38 | Day 42 | Day 45 | Day 48 |
| --- | --- | --- | --- | --- | --- | --- | --- | --- | --- | --- | --- | --- | --- | --- | --- | --- |
| LFD | Mean | 24.48 | 24.16 | 24.16 | 24.58 | 24.52 | 24.91 | 24.73 | 24.70 | 24.80 | 25.13 | 25.09 | 24.98 | 25.55 | 25.27 | 25.92 |
|  | SD | 1.22 | 1.42 | 1.38 | 1.27 | 1.49 | 1.59 | 1.75 | 1.55 | 1.49 | 1.64 | 1.52 | 1.62 | 1.81 | 1.78 | 1.70 |
| HFD | Mean | 29.89^**^ | 29.94^**^ | 30.58^**^ | 30.64^**^ | 31.92^**^ | 32.67^**^ | 33.99^**^ | 34.09^**^ | 35.06^**^ | 35.11^**^ | 36.42^**^ | 37.09^**^ | 38.59^**^ | 38.07^**^ | 38.76^**^ |
|  | SD | 1.40 | 1.74 | 2.04 | 1.93 | 1.57 | 1.85 | 1.28 | 1.23 | 1.27 | 1.14 | 1.03 | 1.25 | 1.56 | 1.74 | 1.56 |
| Orlistat | Mean | 29.88^**^ | 27.33^**##^ | 25.14^##^ | 24.89^##^ | 25.34^##^ | 26.69^*##^ | 27.52^**##^ | 28.36^**##^ | 28.70^**##^ | 29.06^**##^ | 28.33^**##^ | 29.15^**##^ | 30.95^**##^ | 31.90^**##^ | 32.47^**##^ |
|  | SD | 1.37 | 1.22 | 1.10 | 1.15 | 1.11 | 1.21 | 1.38 | 1.19 | 1.44 | 1.53 | 1.71 | 1.89 | 2.04 | 1.84 | 1.70 |
| CJB38 L | Mean | 29.89^**^ | 29.07^**^ | 29.72^**^ | 29.94^**^ | 30.93^**^ | 31.86^**^ | 32.45^**^ | 32.53^**^ | 33.45^**^ | 34.17^**^ | 34.74^**^ | 35.49^**^ | 36.72^**^ | 36.58^**^ | 36.92^**^ |
|  | SD | 1.39 | 1.42 | 1.67 | 1.72 | 1.70 | 1.70 | 1.90 | 1.88 | 1.98 | 1.81 | 1.76 | 1.78 | 1.97 | 2.00 | 1.80 |
| CJB38 M | Mean | 29.88^**^ | 29.22^**^ | 29.89^**^ | 30.14^**^ | 30.90^**^ | 31.67^**^ | 32.29^**#^ | 32.33^**#^ | 33.17^**#^ | 33.76^**^ | 34.10^**#^ | 34.52^**#^ | 36.30^**^ | 36.09^**^ | 36.14^**#^ |
|  | SD | 1.39 | 1.22 | 1.23 | 1.34 | 1.60 | 1.92 | 2.08 | 2.10 | 2.17 | 2.41 | 2.76 | 3.12 | 3.55 | 3.36 | 3.44 |
| CJB38 H | Mean | 29.87^**^ | 28.89^**^ | 29.51^**^ | 30.20^**^ | 30.87^**^ | 31.61^**^ | 32.28^**#^ | 32.33^**#^ | 33.14^**#^ | 33.84^**^ | 34.81^**^ | 35.43^**^ | 37.05^**^ | 36.85^**^ | 36.97^**^ |
|  | SD | 1.39 | 1.56 | 1.47 | 1.50 | 1.59 | 1.87 | 2.20 | 2.20 | 2.46 | 2.58 | 2.88 | 3.05 | 3.29 | 3.71 | 3.32 |
| CJB46 L | Mean | 29.89^**^ | 29.65^**^ | 30.21^**^ | 30.29^**^ | 31.01^**^ | 31.66^**^ | 32.16^**#^ | 32.33^**#^ | 33.26^**^ | 33.61^**^ | 33.81^**#^ | 34.15^**##^ | 35.54^**#^ | 35.24^**#^ | 35.34^**##^ |
|  | SD | 1.43 | 1.68 | 1.78 | 1.80 | 1.84 | 1.81 | 2.09 | 2.23 | 2.62 | 2.69 | 2.63 | 2.69 | 2.85 | 3.01 | 3.10 |
| CJB46 M | Mean | 29.88^**^ | 29.62^**^ | 29.97^**^ | 30.58^**^ | 31.20^**^ | 32.05^**^ | 32.36^**#^ | 32.43^**#^ | 33.10^**#^ | 33.43^**^ | 34.09^**#^ | 34.40^**#^ | 35.38^**##^ | 35.15^**#^ | 35.46^**##^ |
|  | SD | 1.39 | 1.41 | 1.63 | 1.62 | 1.53 | 1.59 | 1.79 | 1.49 | 1.77 | 1.96 | 1.95 | 2.01 | 1.93 | 2.06 | 2.12 |
| CJB46 H | Mean | 29.88^**^ | 29.45^**^ | 29.96^**^ | 30.47^**^ | 31.33^**^ | 32.73^**^ | 33.08^**^ | 32.80^**^ | 33.50^**^ | 34.10^**^ | 34.48^**#^ | 34.58^**#^ | 35.69^**#^ | 35.92^**#^ | 36.04^**#^ |
|  | SD | 1.40 | 1.26 | 1.24 | 1.10 | 0.69 | 0.64 | 1.09 | 1.64 | 2.08 | 2.16 | 2.46 | 2.95 | 3.18 | 2.88 | 3.63 |
| CJLS03 L | Mean | 29.89^**^ | 28.70^**^ | 29.00^**#^ | 29.73^**^ | 31.16^**^ | 32.18^**^ | 32.76^**^ | 32.70^**^ | 33.19^**#^ | 33.98^**^ | 34.56^**^ | 35.29^**^ | 36.47^**^ | 36.37^**^ | 36.46^**#^ |
|  | SD | 1.35 | 1.84 | 2.14 | 2.05 | 1.84 | 1.67 | 1.60 | 1.61 | 1.54 | 1.72 | 1.69 | 1.70 | 1.92 | 2.05 | 2.01 |
| CJLS03 M | Mean | 29.88^**^ | 29.03^**^ | 29.15^**#^ | 29.60^**^ | 30.01^**##^ | 30.82^**#^ | 31.27^**##^ | 31.28^**##^ | 31.79^**##^ | 32.43^**#^ | 33.15^**##^ | 33.36^**##^ | 34.42^**##^ | 34.21^**##^ | 34.43^**##^ |
|  | SD | 1.45 | 1.64 | 1.73 | 1.86 | 2.26 | 2.37 | 2.47 | 2.28 | 2.49 | 2.46 | 2.76 | 2.92 | 3.15 | 3.17 | 3.05 |
| CJLS03 H | Mean | 29.87** | 28.69^**^ | 28.36^**##^ | 28.62^**##^ | 29.08^**##^ | 29.85^**##^ | 30.04^**##^ | 30.33^**##^ | 30.88^**##^ | 31.33^**##^ | 31.48^**##^ | 31.44^**##^ | 32.54^**##^ | 32.70^**##^ | 32.75^**##^ |
|  | SD | 1.39 | 1.59 | 1.63 | 1.27 | 1.33 | 1.60 | 1.90 | 1.97 | 2.39 | 2.45 | 2.86 | 2.97 | 2.91 | 3.07 | 2.81 |
