# Supplementary material for: Dose-dependent and strain-dependent anti-obesity effects of Lactobacillus sakei in a diet induced obese murine model
Source: PeerJ. 2019 Mar 21;7:e6651. doi: 10.7717/peerj.6651 (PMC6431538; doi:10.7717/peerj.6651)
Supplement: Supplemental Information 4 — Reduced weight gain effect of different microorganisms administered in a diet induced obesity mouse model after 8 weeks study period. After 1 week of acclimatization, 120 of 6 weeks old male C57/BL6 mice were divided into 12 different groups receiving a low-fat diet with 300 μL PBS as carrier (LFD), high-fat diet with 300 μL PBS (HFD), high-fat diet with 80 mpk of orlistat. The other 9 groups received a high-fat diet with 1x10^9^ CFU of either Lactobacillus plantarum 301 (LP 301), L. sakei CJLS03 (LS 03), L. sakei LS 338, L. sakei LS 446, L. brevis LB 29, L. brevis LB 33, L. brevis LB 54, L. brevis LB 55 or B. longum BL 83. Feed and sterilized water supply were provided ad libitum and weight gains were measured by subtracting the average weight of week 0 from that of week 8. Asterisks denote the level of significance (Dunnett’s multiple comparison test) compared to HFD as *: p<0.05, **: p<0.01 and ***: p<0.001. [file peerj-07-6651-s004.docx]

**Supplementary information**

**Figure S1**

**Dose dependent and strain-dependent anti-obesity effects of *Lactobacillus sakei* in a diet induced obese murine model**

Yosep Ji^1*^, Young Mee Chung^2*^, Soyoung Park^1*^, Dahye Jeong^2^, Bongjoon Kim^2^, Wilhelm H. Holzapfel^1^

^1^Department of Advanced Green Energy and Environment, Handong Global University, Pohang, Gyungbuk 37554, South Korea;

^2^Beneficial microbes center, CJ Foods R&D, CJ CheilJedang Corporation, Suwon-si, SouthKorea


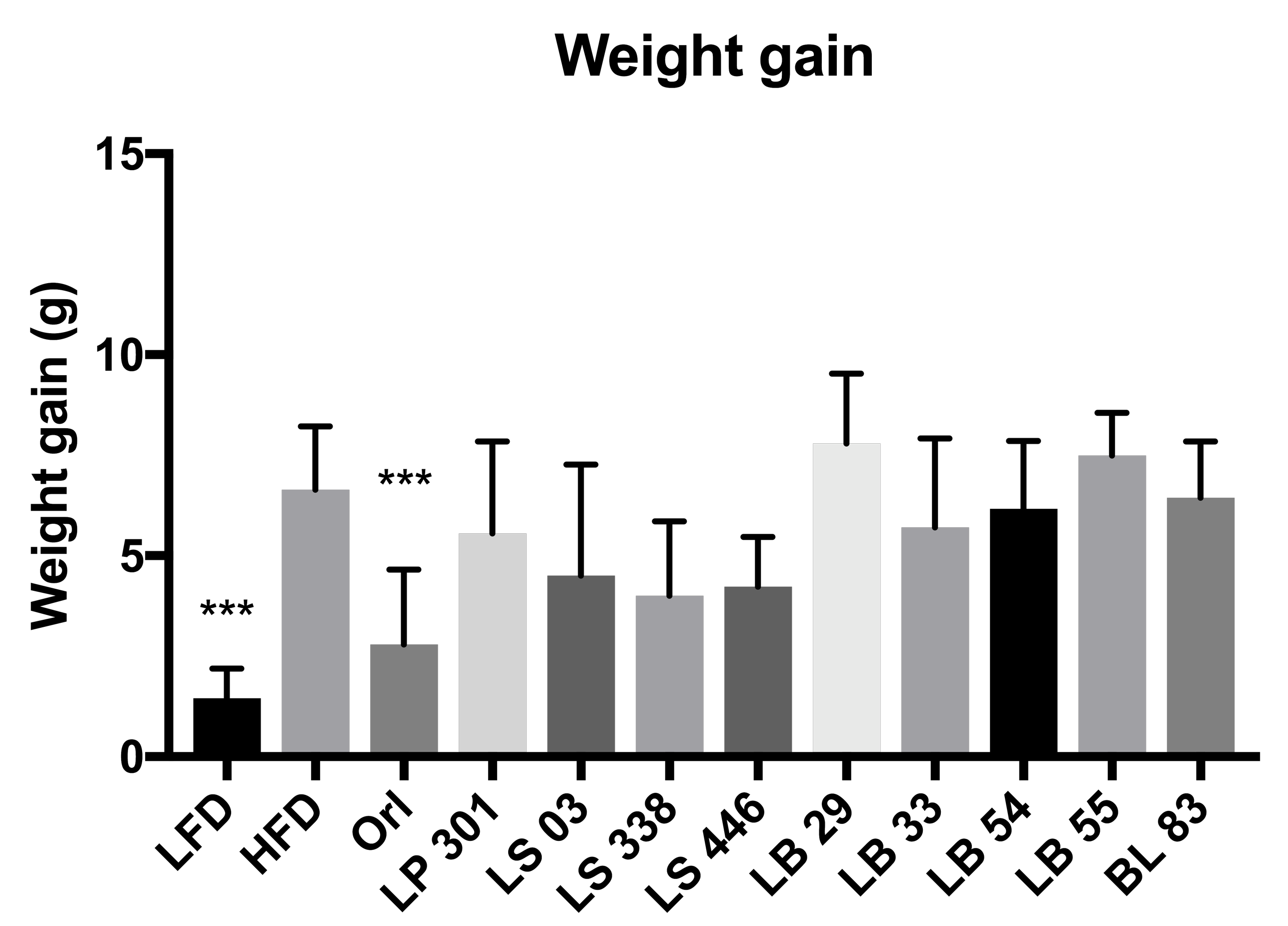


**Supplementary Figure S1.** Reduced weight gain effect of different microorganisms administered in a diet induced obesity mouse model after 8 weeks study period. After 1 week of acclimatization, 120 of 6 weeks old male C57/BL6 mice were divided into 12 different groups receiving a low-fat diet with 300 μL PBS as carrier (LFD), high-fat diet with 300 μL PBS (HFD), high-fat diet with 80 mpk of orlistat. The other 9 groups received a high-fat diet with 1x10^9^ CFU of either *Lactobacillus plantarum* 301 (LP 301), *L. sakei* CJLS03 (LS 03), *L. sakei* LS 338, *L. sakei* LS 446, *L. brevis* LB 29, *L. brevis* LB 33, *L. brevis* LB 54, *L. brevis* LB 55 or *B. longum* BL 83. Feed and sterilized water supply were provided *ad libitum* and weight gains were measured by subtracting the average weight of week 0 from that of week 8. Asterisks denote the level of significance (Dunnett’s multiple comparison test) compared to HFD as *: p<0.05, **: p<0.01 and ***: p<0.001.
