# Supplementary material for: Dose-dependent and strain-dependent anti-obesity effects of Lactobacillus sakei in a diet induced obese murine model
Source: PeerJ. 2019 Mar 21;7:e6651. doi: 10.7717/peerj.6651 (PMC6431538; doi:10.7717/peerj.6651)
Supplement: Supplemental Information 5 — Equation, growth curves and plate counting results for L. sakei LS03, L. sakei L338 and L. sakei L446. [file peerj-07-6651-s005.docx]

**Supplementary information**

**Figure S2**

**Dose dependent and strain-dependent anti-obesity effects of *Lactobacillus sakei* in a diet induced obese murine model**

Yosep Ji^1*^, Young Mee Chung^2*^, Soyoung Park^1*^, Dahye Jeong^2^, Bongjoon Kim^2^, Wilhelm H. Holzapfel^1^

^1^Department of Advanced Green Energy and Environment, Handong Global University, Pohang, Gyungbuk 37554, South Korea;

^2^Beneficial microbes center, CJ Foods R&D, CJ CheilJedang Corporation, Suwon-si, South Korea

|  | ***L. sakei* LS03** | | ***L. sakei* L338** | | ***L. sakei* L446** | |
| --- | --- | --- | --- | --- | --- | --- |
| **Growth curve** |  | |  | |  | |
| **Plate counting results** | **hours** | **CFU (log)** | **hours** | **CFU (log)** | **hours** | **CFU (log)** |
|  | 13 | 8.03 | 8 | 8.08 | 8 | 8.19 |
|  | 11 | 7.48 | 6 | 7.74 | 6 | 7.82 |
|  | 9 | 6.77 | 4 | 7.11 | 4 | 6.81 |
|  | 7 | 6.45 | 2 | 6.79 | 2 | 5.90 |
| **Equation** | y = 0.626x+5.782  (y = CFU in log; x = O.D) | | y = 0.4054x+6.3589  (y = CFU in log; x = O.D) | | y = 0.7675x+4.9868  (y = CFU in log; x = O.D) | |

**Supplementary Figure 2** Equation, growth curves and plate counting results for *L. sakei* LS03, *L. sakei* L338 and *L. sakei* L446.
