# Supplementary material for: Dose-dependent and strain-dependent anti-obesity effects of Lactobacillus sakei in a diet induced obese murine model
Source: PeerJ. 2019 Mar 21;7:e6651. doi: 10.7717/peerj.6651 (PMC6431538; doi:10.7717/peerj.6651)
Supplement: Supplemental Information 6 — Dose dependent anti-obesity effects of CJLS03 with regard to weight gain, serum biomarkers and adipose tissue weight. All data were measured at the end of the experimental period (at 48 days) and RE expressed as mean value (n=10). LFD, normal diet (low-fat diet); HFD, high-fat diet; OLS, Orlistat 40 mg/kg. L (high-dose), 1 X 1010 CFU/mL; M (medium-dose), 1 X 109 CFU/mL; L (low-dose), 1 X 108 CFU/mL. TG, serum triglycerides; TC, total cholesterol; AST, aspartate transaminase. [file peerj-07-6651-s006.docx]

**Supplementary information**

**Figure S3**

**Dose dependent and strain-dependent anti-obesity effects of *Lactobacillus sakei* in a diet induced obese murine model**

Yosep Ji^1*^, Young Mee Chung^2*^, Soyoung Park^1*^, Dahye Jeong^2^, Bongjoon Kim^2^, Wilhelm H. Holzapfel^1^

^1^Department of Advanced Green Energy and Environment, Handong Global University, Pohang, Gyungbuk 37554, South Korea;

^2^Beneficial microbes center, CJ Foods R&D, CJ CheilJedang Corporation, Suwon-si, South Korea


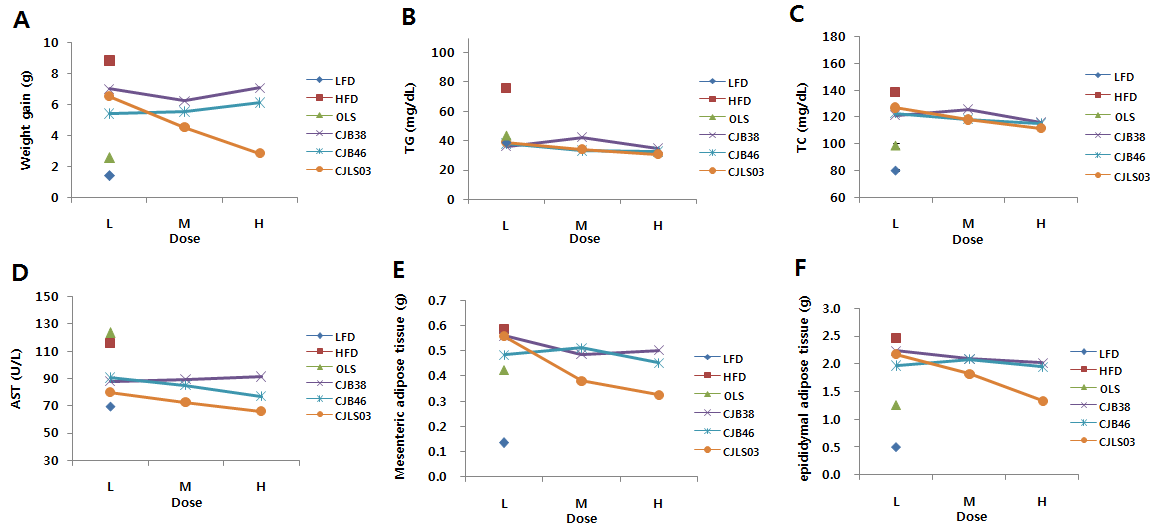


**Supplementary Figure S3** Dose dependent anti-obesity effects of CJLS03 with regard to weight gain, serum biomarkers and adipose tissue weight. All data were measured at the end of the experimental period (at 48 days) and RE expressed as mean value (n=10). LFD, normal diet (low-fat diet); HFD, high-fat diet; OLS, Orlistat 40 mg/kg. L (high-dose), 1 X 10^10^ CFU/mL; M (medium-dose), 1 X 10^9^ CFU/mL; L (low-dose), 1 X 10^8^ CFU/mL. TG, serum triglycerides; TC, total cholesterol; AST, aspartate transaminase.
