# Supplementary material for: Dose-dependent and strain-dependent anti-obesity effects of Lactobacillus sakei in a diet induced obese murine model
Source: PeerJ. 2019 Mar 21;7:e6651. doi: 10.7717/peerj.6651 (PMC6431538; doi:10.7717/peerj.6651)
Supplement: Supplemental Information 7 — Histological analyses of hepatic lipid accumulation. Representative HE-stained liver sections are shown (magnification, 200X). Mice fed a high-fat diet showed considerable hepatic lipid accumulation compared with normal pellet diet. Moderate vacuolations around the portal triad are shown in the HFD-fed mouse. LFD, normal diet (low-fat diet); HFD, high-fat diet; OLS, Orlistat 40 mg/kg; CJB38H, CJB46H, and CJLS03H received 1 X 1010 CFUs respectively of the L. sakei strains CJB38, CJB46, and CJLS03. [file peerj-07-6651-s007.docx]

**Supplementary information**

**Figure S4**

**Dose dependent and strain-dependent anti-obesity effects of *Lactobacillus sakei* in a diet induced obese murine model**

Yosep Ji^1*^, Young Mee Chung^2*^, Soyoung Park^1*^, Dahye Jeong^2^, Bongjoon Kim^2^, Wilhelm H. Holzapfel^1^

^1^Department of Advanced Green Energy and Environment, Handong Global University, Pohang, Gyungbuk 37554, South Korea;

^2^Beneficial microbes center, CJ Foods R&D, CJ CheilJedang Corporation, Suwon-si, South Korea

**
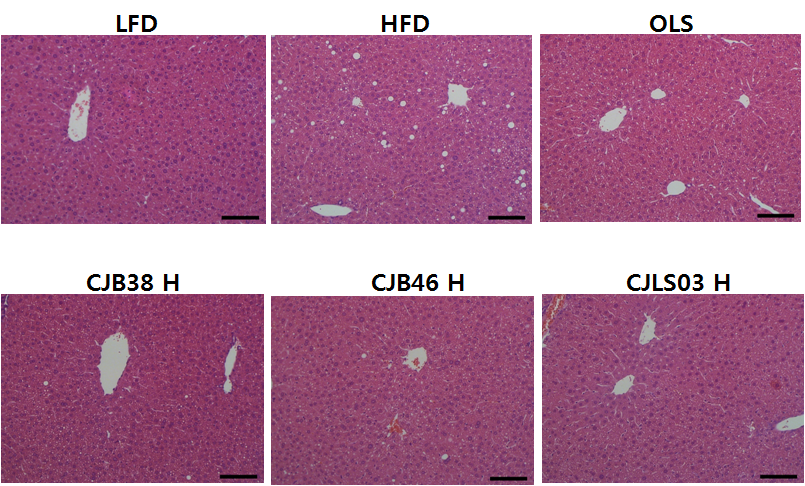
**

**Supplementary Figure S4.** Histological analyses of hepatic lipid accumulation. Representative HE-stained liver sections are shown (magnification, 200X). Mice fed a high-fat diet showed considerable hepatic lipid accumulation compared with normal pellet diet. Moderate vacuolations around the portal triad are shown in the HFD-fed mouse. LFD, normal diet (low-fat diet); HFD, high-fat diet; OLS, Orlistat 40 mg/kg; CJB38H, CJB46H, and CJLS03H received 1 X 10^10^ CFUs respectively of the *L. sakei* strains CJB38, CJB46, and CJLS03.
